# Vitamin D and COVID-19 infection and mortality in UK Biobank

**DOI:** 10.1007/s00394-020-02372-4

**Published:** 2020-08-26

**Authors:** Claire E. Hastie, Jill P. Pell, Naveed Sattar

**Affiliations:** 1 Institute of Health and Wellbeing, University of Glasgow, 1 Lilybank Gardens, Glasgow, G12 8RZ UK; 2 Institute of Cardiovascular and Medical Sciences, University of Glasgow, Glasgow, UK

**Keywords:** COVID-19, Vitamin D, Mortality

## Abstract

**Purpose:**

Low blood 25-hydroxyvitamin D (25(OH)D) concentration has been proposed as a potential causal factor in COVID-19 risk. We aimed to establish whether baseline serum 25(OH)D concentration was associated with COVID-19 mortality, and inpatient confirmed COVID-19 infection, in UK Biobank participants.

**Methods:**

UK Biobank recruited 502,624 participants aged 37–73 years between 2006 and 2010. Baseline exposure data, including serum 25(OH)D concentration, were linked to COVID-19 mortality. Univariable and multivariable Cox proportional hazards regression analyses were performed for the association between 25(OH)D and COVID-19 death, and Poisson regression analyses for the association between 25(OH)D and severe COVID-19 infection.

**Results:**

Complete data were available for 341,484 UK Biobank participants, of which 656 had inpatient confirmed COVID-19 infection and 203 died of COVID-19 infection. 25(OH)D concentration was associated with severe COVID-19 infection and mortality univariably (mortality per 10 nmol/L 25(OH)D HR  0.92; 95% CI 0.86–0.98; *p* = 0.016), but not after adjustment for confounders (mortality per 10 nmol/L 25(OH)D HR 0.98; 95% CI = 0.91–1.06; *p* = 0.696). Vitamin D insufficiency or deficiency was also not independently associated with either COVID-19 infection or linked mortality.

**Conclusions:**

Our findings do not support a potential link between 25(OH)D concentrations and risk of severe COVID-19 infection and mortality. Randomised trials are needed to prove a beneficial role for vitamin D in the prevention of severe COVID-19 reactions or death.

In the hunt for modifiable COVID-19 risk factors, vitamin D has gained a lot of attention both in the media and within the scientific community [1]. Proponents of such a link cite a few early studies that present circumstantial evidence. They are either ecological, at an individual level but unable to fully adjust for potential confounders, or they measured 25-hydroxyvitamin D (25(OH)D) concentration once patients were already hospitalised with COVID-19 which introduces reverse causation, as vitamin D is a negative acute phase reactant [2].

Despite the sparse evidence on vitamin D in COVID-19 [3], the UK government led an urgent review into whether there is any link. It concluded that “There is no evidence to support taking vitamin D supplements to specifically prevent or treat COVID-19” [4]. By contrast, the Welsh COVID-19 risk assessment tool includes vitamin D supplementation as part of its recommendations [5]. Furthermore, a recent review conducted by the Scientific Advisory Committee on Nutrition found insufficient evidence to support recommending vitamin D supplementation to prevent acute respiratory tract infections in the general UK population [6].

We previously observed no evidence of an association between baseline serum 25(OH)D concentration and testing positive for SARS-CoV-2 in hospital in UK Biobank once potential confounders were adjusted for [7]. Importantly, some of the variables that were associated with increased COVID-19 risk in our sample, for example lower socioeconomic status, being Black or South Asian, or being overweight or obese, are also associated with lower vitamin D. This suggests that the positive findings of other studies may in part be due to inadequate adjustment. Another recent study of UK Biobank data replicated this finding [8], but it would be more informative to relate 25(OH)D concentration to COVID-19-related mortality.

In the current analysis, we therefore linked baseline serum 25(OH)D concentration in 341,484 UK Biobank participants with complete data on covariates to Death Register data. In the sample, 203 participants died due to COVID-19 infection. Deaths occurred between the 5th of March and 25th of April 2020. We explored whether serum 25(OH)D concentration as a continuous measurement, or vitamin D deficiency or insufficiency (defined as serum 25(OH)D < 25 and < 50 nmol/L, respectively), were associated with risk of COVID-19 death using Cox proportional hazards regression analysis.

Univariably, several covariates were associated with COVID-19 mortality and infection (Table 1). Notably, black and South Asian ethnicity, obesity, and lower socioeconomic status are also associated with lower 25(OH)D concentration.

**Table 1:** Univariable association between baseline covariates and confirmed COVID-19 mortality, and confirmed inpatient COVID-19 infection

	COVID-19 mortality		Inpatient COVID-19 infection	
	HR (95% CI)	*p*	IRR (95% CI)	*p*
Sex				
Female	1		1	
Male	2.49 (1.85–3.35)	< 0.001	1.50 (1.29–1.75)	< 0.001
Self-reported ethnicity				
White	1		1	
Black	7.44 (4.30–12.9)	< 0.001	3.14 (2.17–4.53)	< 0.001
South Asian	1.76 (0.65–4.74)	0.264	2.69 (1.87–3.89)	< 0.001
Townsend deprivation quintile				
1	1		1	
2	1.05 (0.64–1.72)	0.855	1.04 (0.79–1.36)	0.802
3	0.95 (0.57–1.58)	0.845	1.08 (0.82–1.42)	0.584
4	1.47 (0.93–2.34)	0.101	1.37 (1.06–1.77)	0.016
5	2.56 (1.67–3.91)	< 0.001	2.23 (1.75–2.83)	< 0.001
Household income				
< £18,000	1		1	
£18 k-£30,999	0.47 (0.33–0.67)	< 0.001	0.62 (0.51–0.76)	< 0.001
£31 k-£51,999	0.42 (0.29–0.60)	< 0.001	0.55 (0.45–0.68)	< 0.001
£52 k-£100,000	0.28 (0.18–0.44)	< 0.001	0.44 (0.34–0.55)	< 0.001
> £100,000	0.14 (0.04–0.43)	0.001	0.36 (0.23–0.56)	< 0.001
Overall health rating				
Excellent	1		1	
Good	1.52 (0.94–2.43)	0.085	1.49 (1.15–1.93)	0.003
Fair	3.49 (2.14–5.68)	< 0.001	2.46 (1.86–3.26)	< 0.001
Poor	4.86 (2.59–9.12)	< 0.001	4.97 (3.54–6.96)	< 0.001
Long-standing illness, disability or infirmity				
No	1		1	
Yes	2.80 (2.13–3.70)	< 0.001	1.84 (1.58–2.15)	< 0.001
Smoking status				
No	1		1	
Yes	1.36 (0.91–2.02)	0.134	1.34 (1.07–1.69)	0.013
BMI category				
Normal weight	1		1	
Underweight	1.86 (0.26–13.6)	0.539	1.38 (0.44–4.31)	0.584
Overweight	1.82 (1.23–2.67)	0.002	1.50 (1.23–1.83)	< 0.001
Obese	3.13 (2.12–4.62)	< 0.001	2.02 (1.64–2.49)	< 0.001
Diabetes				
No	1		1	
Yes	5.06 (3.59–7.13)	< 0.001	2.49 (1.96–3.17)	< 0.001
Current age (years)	1.13 (1.11–1.16)	< 0.001	1.01 (1.00–1.02)	0.007
Baseline systolic blood pressure (mmHg)	1.02 (1.02–1.03)	< 0.001	1.00 (0.999–1.01)	0.123
Baseline diastolic blood pressure (mmHg)	1.01 (0.998–1.03)	0.099	1.01 (0.999–1.01)	0.109

*HR* hazard ratio, *CI* confidence interval, *IRR* incidence rate ratio

The COVID-19 mortality results followed the same pattern that we previously observed for COVID-19 infection [7]. Lower 25(OH)D concentration and vitamin D deficiency were both associated with higher risk of COVID-19 death univariably, but not after adjustment for potential confounders (Table 2). Multivariate models were adjusted for all measured confounders as detailed in the table legend. Vitamin D insufficiency was not associated with risk of COVID-19 death univariably or multivariably. Furthermore, we repeated our previous analysis of the association between 25(OH)D and confirmed COVID-19 infection with additional cases, using univariable and multivariable poisson regression of inpatient diagnosed infection. There were 656 confirmed inpatient COVID-19 cases. Again, 25(OH)D concentration and vitamin D deficiency were associated with COVID-19 infection univariably but not multivariably (Table 2).

**Table 2:** Association between baseline serum 25(OH)D and confirmed COVID-19 mortality, and confirmed inpatient COVID-19 infection

	COVID-19 mortality				Inpatient COVID-19 infection			
	Univariable		Multivariable*		Univariable		Multivariable*	
	HR (95% CI)	*p* value	HR (95% CI)	*p* value	IRR (95% CI)	*p* value	IRR (95% CI)	*p* value
25(OH)D (per 10 nmol/L)	0.92 (0.86–0.98)	0.016	0.98 (0.91–1.06)	0.696	0.93 (0.90–0.97)	< 0.001	1.00 (0.96–1.05)	0.888
Vitamin D deficient (25(OH)D < 25 nmol/L)	1.61 (1.14–2.27)	0.007	1.21 (0.83–1.76)	0.311	1.56 (1.28–1.90)	< 0.001	1.10 (0.88–1.37)	0.404
Vitamin D insufficient (25(OH)D < 50 nmol/L)	1.29 (0.97–1.72)	0.076	1.02 (0.75–1.38)	0.919	1.33 (1.14–1.56)	< 0.001	1.06 (0.89–1.26)	0.525

Participants who died of COVID-19 had a median age at death of 76 years (interquartile range 71–78 years)

*HR* hazard ratio, *CI* confidence interval, *IRR* incidence rate ratio

*Adjusted for age, sex, ethnicity, month of assessment, Townsend deprivation quintile, household income, BMI category, smoking status, diabetes, systolic blood pressure, diastolic blood pressure, self-reported health rating, and long-standing illness, disability or infirmity

The variables significantly associated with risk of COVID-19 mortality in multivariate analysis were age (HR 1.12; 95% CI 1.10–1.15; *p* < 0.001 per year), male sex (HR 2.12; 95% CI 1.56–2.89; *p* < 0.001), black ethnicity (HR 8.13; 95% CI 4.56–14.50; *p* < 0.001), obesity (HR 1.68; 95% CI 1.11–2.56; *p* = 0.015 compared with normal weight), socioeconomic deprivation (highest Townsend deprivation quintile compared with lowest HR 1.96; 95% CI 1.24–3.09; *p* = 0.004), and diabetes (HR 1.96; 95% CI 1.34–2.86; *p* = 0.001). These findings are consistent with other studies, lending strong external validity.

The main limitation of using UK Biobank for this analysis is the ~ 10 year time period between baseline 25(OH)D measurement and COVID-19 infection. We examined the concordance rates of vitamin D deficiency in a subsample of 15,473 participants who had measurements taken both at baseline and at a follow-up visit (on average 4.3 years later). Concordance in this group was 84%.

If there is a causal link, vitamin D supplements would present an appealingly cheap low risk intervention. However, currently there is no evidence that supplements will reduce risk of COVID-19 infection [4], or acute respiratory tract infections more generally [6]. NHS guidelines already recommend that all UK residents take vitamin D supplements in the winter, and furthermore that certain groups who are more likely to be deficient (for example those with darker skin) take them throughout the year [9]. We await the results of randomised controlled trials to determine whether there should be any change to these guidelines and consequently clinical practice. For now, recommendations for vitamin D supplementation to lessen COVID-19 risks appear premature and, although they may cause little harm, they could provide false reassurance leading to changes in behaviour that increase risk of infections.

## Data Availability

All analyses were undertaken using Stata v14.

## Availability of data and material

Data can be accessed via the procedures detailed in the UK Biobank website (https://www.ukbiobank.ac.uk/).

## References

[1] Mitchell F (2020). Vitamin-D and COVID-19: do deficient risk a poorer outcome?. Lancet Diabetes Endocrinol.

[2] Sattar N, Welsh P, Panarelli M, Forouhi NG (2012). Increasing requests for vitamin D measurement: costly, confusing, and without credibility. Lancet.

[3] The Centre for Evidence-Based Medicine. (2020) Vitamin D: a rapid review of the evidence for treatment or prevention in COVID-19. https://www.cebm.net/covid-19/vitamin-d-a-rapid-review-of-the-evidence-for-treatment-or-prevention-in-covid-19/. Accessed 26 Jun 2020

[4] NICE. COVID-19 rapid evidence summary:vitamin D for COVID-19. https://www.nice.org.uk/advice/es28/evidence. Accessed 11 Aug 2020.

[5] (2020) Welsh Government. COVID-19 workforce risk assessment tool. https://gov.wales/checkyourrisk. Accessed 11 Aug 2020

[6] Scientific Advisory Committee on Nutrition. Vitamin D and acute respiratory tract infections. https://app.box.com/s/g0ldpth1upfd7fw763ew3aqa3c0pyvky. Accessed 11 Aug 2020.

[7] Hastie CE, Mackay DF, Ho F (2020). Vitamin D concentrations and COVID-19 infection in UK Biobank. Diabetes Metab Syndr Clin Res Rev.

[8] Raisi-Estabragh Z, McCracken C, Bethell MS (2020). Greater risk of severe COVID-19 in Black, Asian and Minority Ethnic populations is not explained by cardiometabolic, socioeconomic or behavioural factors, or by 25(OH)-vitamin D status: study of 1326 cases from the UK Biobank. J Public Heal.

[9] NHS. Vitamins and minerals: Vitamin D. https://www.nhs.uk/conditions/vitamins-and-minerals/vitamin-d/. Accessed 26 Jun 2020

